# Validation and Selection of New Reference Genes for RT-qPCR Analysis in Pediatric Glioma of Different Grades

**DOI:** 10.3390/genes12091335

**Published:** 2021-08-27

**Authors:** Beatriz Hernández-Ochoa, Fabiola Fernández-Rosario, Rosa Angelica Castillo-Rodríguez, Alfonso Marhx-Bracho, Noemí Cárdenas-Rodríguez, Víctor Martínez-Rosas, Laura Morales-Luna, Abigail González-Valdez, Ernesto Calderón-Jaimes, Verónica Pérez de la Cruz, Sandra Rivera-Gutiérrez, Sergio Meza-Toledo, Carlos Wong-Baeza, Isabel Baeza-Ramírez, Saúl Gómez-Manzo

**Affiliations:** 1 Programa de Posgrado en Biomedicina y Biotecnología Molecular, Escuela Nacional de Ciencias Biológicas, Instituto Politécnico Nacional, Ciudad de México 11340, Mexico; beatrizhb_16@hotmail.com (B.H.-O.); ing_vicmr@hotmail.com (V.M.-R.); 2 Laboratorio de Inmunoquímica, Hospital Infantil de México Federico Gómez, Secretaría de Salud, Ciudad de México 06720, Mexico; ecalderj5@yahoo.com.mx; 3 Laboratorio de Bioquímica Genética, Instituto Nacional de Pediatría, Secretaría de Salud, Ciudad de México 04530, Mexico; faby.fernandez.ross@gmail.com (F.F.-R.); lauraeloisamorales@ciencias.unam.mx (L.M.-L.); 4 Consejo Nacional de Ciencia y Tecnología (CONACYT), Instituto Nacional de Pediatría, Secretaría de Salud, Ciudad de México 04530, Mexico; racastilloro@conacyt.mx; 5 Departamento de Neurocirugía, Instituto Nacional de Pediatría, Secretaría de Salud, Ciudad de México 04530, Mexico; marhxalfons@yahoo.com.mx; 6 Laboratorio de Neurociencias, Instituto Nacional de Pediatría, Secretaría de Salud, Ciudad de México 04530, Mexico; noemicr2001@yahoo.com.mx; 7 Posgrado en Ciencias Biológicas, Universidad Nacional Autónoma de México, Ciudad de México 04510, Mexico; 8 Departamento de Biología Molecular y Biotecnología, Instituto de Investigaciones Biomédicas, Universidad Nacional Autónoma de México, Ciudad de México 04510, Mexico; abigaila@iibiomedicas.unam.mx; 9 Neurochemistry and Behavior Laboratory, National Institute of Neurology and Neurosurgery “Manuel Velasco Suárez”, México City 14269, Mexico; veped@yahoo.com.mx; 10 Departamento de Microbiologia, Escuela Nacional de Ciencias Biológicas (ENCB), Instituto Politécnico Nacional (IPN), Prolongacion Carpio y Plan de Ayala s/n, Ciudad de México 11340, Mexico; san_rg@yahoo.com.mx; 11 Laboratorio de Quimioterapia Experimental, Departamento de Bioquímica, Escuela Nacional de Ciencias Biológicas, Instituto Politécnico Nacional, Ciudad de México 11340, Mexico; semeza@hotmail.com; 12 Laboratorio de Biomembranas, Departamento de Bioquímica, Escuela Nacional de Ciencias Biológicas, Instituto Politécnico Nacional, Ciudad de México 11340, Mexico; charlywong@icloud.com (C.W.-B.); isabelbaeza@yahoo.com (I.B.-R.)

**Keywords:** references genes, real-time quantitative PCR, gene stability, gliomas

## Abstract

Gliomas are heterogeneous, solid, and intracranial tumors that originate from glial cells. Malignant cells from the tumor undergo metabolic alterations to obtain the energy required for proliferation and the invasion of the cerebral parenchyma. The alterations in the expression of the genes related to the metabolic pathways can be detected in biopsies of gliomas of different CNS WHO grades. In this study, we evaluated the expression of 16 candidate reference genes in the HMC3 microglia cell line. Then, statistical algorithms such as BestKeeper, the comparative ΔC_T_ method, geNorm, NormFinder, and RefFinder were applied to obtain the genes most suitable to be considered as references for measuring the levels of expression in glioma samples. The results show that *PKM* and *TPI1* are two novel genes suitable for genic expression studies on gliomas. Finally, we analyzed the expression of genes involved in metabolic pathways in clinical samples of brain gliomas of different CNS WHO grades. RT-qPCR analysis showed that in CNS WHO grade 3 and 4 gliomas, the expression levels of *HK1*, *PFKM*, *GAPDH*, *G6PD*, *PGD1*, *IDH1*, *FASN*, *ACACA*, and *ELOVL2* were higher than those of CNS WHO grade 1 and 2 glioma biopsies. Hence, our results suggest that reference genes from metabolic pathways have different expression profiles depending on the stratification of gliomas and constitute a potential model for studying the development of this type of tumor and the search for molecular targets to treat gliomas.

## 1. Introduction

Glioma is the most common and lethal tumors of the central nervous system (CNS); in the USA alone, an incidence rate of 3.22 per 100,000 population is reported [1]. The World Health Organization (WHO, Geneva, Switzerland) classified gliomas as either grade 1 tumors, which are curable and can be surgically removed; lower-grade gliomas correspond to grade 2; and malignant gliomas are classified as either grade 3 tumors such as anaplastic astrocytoma or grade 4 tumors such as glioblastoma multiforme (GBM) [2].

Gliomas account for approximately 55% of malignant primary brain tumors, and in approximately 70% of cases, the glioma is classified as an astrocytoma CNS WHO grade 4 (GBM) [3]—a very aggressive tumor. Patients have an expected survival of only a year after GBM diagnosis due to the proliferative, invasive, and infiltrative nature of GBM cells, and also the resistance of these cells to current cytotoxic treatment based on the use of temozolomide [4,5]. Therefore, it is necessary to identify and implement new predictive markers that will help us to perform early diagnosis and better treatment. As a matter of fact, the use of gene expression analysis combined with clinical data has allowed better diagnosis and a better prognosis for response to cancer therapy [6,7]. However, despite intense medical efforts, there is a lack of definitive information on the etiology of malignant gliomas. Therefore, the identification and implementation of new markers that could help to diagnose, predict, and treat gliomas is an urgent priority. The real-time reverse transcription-quantitative PCR (RT-qPCR) technique has been used in several cancer studies to identify specific molecular targets in tumors [8,9]. Building on these successes, attempts are being made to identify specific molecular markers for brain glioma treatment and identify new specific therapeutic targets for tumor subtypes.

The RT-qPCR technique has become the method of choice for the quantification of accurate gene expression [10]. The advantages of this procedure over other methods for measuring messenger RNA (mRNA) levels include the characteristics that it is easy to perform, sensitive, specific, and reproducible [11,12]. Besides these advantages, it is very sensitive and allows researchers to measure quite low expression levels of mRNA; thus, this technique has become an essential method in research as it allows the reliable quantification of transcripts [13]. Nevertheless, the validity of the obtained results depends on a careful and rigorous selection of reference genes that are characterized by high stability and low expression variability regardless of the tissue or cell type, stage of the disease, experimental development, or treatment [14].

Among the most common genes used as reference genes in RT-qPCR studies are glyceraldehyde-3-phosphate dehydrogenase (*GAPDH*), β actin (*ACTB*), β glucuronidase (*GUSB*), hypoxanthine guanine phosphoribosyl transferase (*HPRT1*), hydroxymethylbilane synthase (*HMBS*), TATA-box binding protein (*TBP*), and small ribosomal subunit (*18S*). However, some studies have reported that these common genes show variability in expression levels in different samples and experimental conditions [15,16]. Based on the above, it is clear that the reference genes are specific for a group of samples and experimental models; thus, the validation and selection of suitable reference genes is essential for reliable analysis in RT-qPCR studies. Although some studies have validated reference genes for the accurate quantification of gene expression in glioma clinical samples, including *TBP* and *HPRT1*, 14-3-3 protein zeta/delta (*YWHAZ*), and 60S ribosomal protein L13a (*RPL13A*) [17,18,19], the reference genes found and reported in these studies are different.

Therefore, the aim of this study was to evaluate the suitability of 16 candidate reference genes: hexokinase 1 (*HK1*), phosphofructokinase muscle (*PFKM*), triosephosphate isomerase 1 (*TPI1*), glyceraldehyde-3-phosphate dehydrogenase (*GAPDH*), pyruvate kinase (*PKM*), lactate dehydrogenase A (*LDHAL6A*), glucose-6-phosphate dehydrogenase (*G6PD*), phosphogluconate dehydrogenase 1 (*PGD1*), transketolase 1 (*TKT1*), succinate dehydrogenase B (*SDHB*), fatty acid synthase (*FASN*), acetyl CoA carboxylase (*ACACA*), fatty acid elongase 2 (*ELOVL2*), and TATA-binding protein (*TBP*) for expression analysis in RT-qPCR in brain glioma biopsies. The MHC3 microglia cell line was used to evaluate candidate reference genes, and stability was analyzed using statistical algorithms such as BestKeeper, the comparative ΔC_T_ method, geNorm, NormFinder, and RefFinder. Furthermore, a gene expression analysis was performed in brain glioma biopsies from pediatric patients, as knowing the alterations in the expression levels of genes that encode proteins that participate in metabolic pathways can provide potentially important clinical outcome markers or targets for therapeutics in gliomas.

Therefore, the aim of this study was to evaluate the suitability of sixteen candidate reference genes: hexokinase 1 (*HK1*), phosphofructokinase muscle (*PFKM*), triosephosphate isomerase 1 (*TPI1*), glyceraldehyde-3-phosphate dehydrogenase (*GAPDH*), pyruvate kinase (*PKM*), lactate dehydrogenase A (*LDHAL6A*), glucose-6-phosphate dehydrogenase (*G6PD*), phosphogluconate dehydrogenase 1 (*PGD1*), transketolase 1 (*TKT1*), succinate dehydrogenase B (*SDHB*), fatty acid synthase (*FASN*), acetyl CoA carboxylase (*ACACA*), fatty acid elongase 2 (*ELOVL2*), and TATA-binding protein (*TBP*) for expression analysis in RT-qPCR in brain glioma biopsies. The MHC3 microglia cell line was used to evaluate candidate reference genes, and stability was analyzed using statistical algorithms, such as BestKeeper, the comparative ΔC_T_ method, geNorm, NormFinder, and RefFinder. Furthermore, a gene expression analysis was performed in brain glioma biopsies from pediatric patients, since knowing the alterations in the expression levels of genes that encode proteins that participate in metabolic pathways can provide potentially important clinical outcome markers or targets for therapeutics in gliomas.

## 2. Materials and Methods

### 2.1. Reference Gene Selection

Reference genes have been identified as those that have functions in essential biological processes including molecular transport, RNA metabolism, oxidative phosphorylation, proteolysis, protein translation, regulation of protein metabolism, and cell cycle control. Based above, sixteen candidate genes encoding enzymes involved in essential metabolic pathways for cell survival such as glycolysis, the pentose phosphate pathway, the Krebs cycle, and fatty acid synthesis (Table 1) were selected to evaluate their suitability as reference genes in gene expression studies by RT-qPCR in brain glioma samples. Six of the genes are involved in glycolysis (*HK1*, *PFKM*, *TPI1*, *GAPDH*, *PKM*, and *LDHAL6A*), three of these genes belong to the pentose phosphate pathway (PPP) (*G6PD*, *PGD1,* and *TKT1*), one gene participates in the Krebs cycle (*SDHB*), and finally, three genes are involved in the fatty acid synthesis pathway (*FASN*, *ACACA,* and *ELOVL2*). Besides, we included three suitable reference genes previously reported as *GAPDH^a^*, *GAPDH^b^*, and *TBP* [17,18,19].

A target screening of our sixteen candidate genes was done using database analysis in silico to reinforce our selection. We searched and selected the GSE16011 glioma database from the Gene Expression Omnibus database (http://www.ncbi.nlm.nih.gov/geo, accessed on 8 July 2021). This database includes 276 glioma samples from grade 1 to grade 4 as well as 8 controls samples [20]. Then, we evaluated the expression of the selected genes using GEO2R online tool (http://www.ncbi.nlm.nih.gov/geo/geo2r/, accessed on 8 July 2021) [21]. We used the Benjamini & Hochberg false discovery rate [22] method to the adjust *p* values and limit the false positives cases, with a cut of *p* < 0.05. The log2-fold change (logFC) for the analyzed genes is showed in Supplementary Table S1. When we compared the glioma samples versus controls, we observed that for all the candidate reference genes selected, the logFC was below 1 or over −1, thus we considered a non-differential expression among glioma versus controls.

Then, the expression values were displayed in a heatmap (Supplementary Figure S1A). A boxplot of the mean expression of these genes is showed comparing glioma samples versus controls (Supplementary Figure S1B). We include *GAPDH* and *TBP* in this analysis and observed a similar tendency that our gene selection. These graphs were obtained using the online software Morpheus (https://software.broadinstitute.org/morpheus/, accessed on 12 July 2021).

### 2.2. Primer Design

The sequences of the primer pairs for the sixteen candidate reference genes used in this study were designed based on the mRNA sequences of the selected genes (Table 2) obtained from the GenBank database (https://www.ncbi.nlm.nih.gov/pmc/, accessed on 11 May 2019). The primers were designed with lengths of 18–22 base pairs (bp) and a mean alignment temperature (Tm) of 60 °C ± 2 °C, and the formation of secondary structures and dimers was verified with the online program OligoEvaluator^TM^ from Sigma Aldrich (http://www.oligoevaluator.com/LoginServlet, accessed on 25 May 2019). The primers were designed to amplify from 180 to 200 bp fragments corresponding to each gene (Table 2). The primer pairs were evaluated using endpoint PCR to analyze their specificity, using synthesized cDNA as a template and the enzyme Q5 High-Fidelity DNA polymerase (New England, BioLabs) with the following amplification conditions: 98 °C for 30 s, 30 cycles at 98 °C for 10 s, 60 °C for 30 s, and 72 °C for 5 min. The PCR products were separated by 2.0% (*w/v*) agarose gel electrophoresis, stained with Midori Green Advance (NIPPON Genetics Europe, Dueren, Germany), and finally analyzed in the MultiDoc-It (UVP) equipment.

### 2.3. Cell Culture

A microglia-derived cell line was used in this study to validate the reference genes, thus the HMC3 cell line was purchased from the American Type Culture Collection (ATCC^®^ CRL-3304^™^). The HMC3 cells were cultured in Eagle’s Minimum Essential Medium (EMEM) and supplemented with 10% fetal bovine serum (FBS; Gibco, Carlsbad, CA, USA) and antibiotics (100 U/mL penicillin and 100 μg/mL streptomycin), according to the manufacturer’s instructions. Cells were grown for 72 h and cultured at 37 °C, 5% CO_2_, in a humidified atmosphere. The experiments were carried out with cultures in the log phase of growth (before the culture reached the monolayer).

### 2.4. Tumor Samples

In this study, 7 samples of brain glioma biopsies were obtained with previous signed informed consent from pediatric patients at the Neurosurgery Department of the Instituto Nacional de Pediatria in Mexico City between November 2018 and August 2019. The protocol was approved by the Institutional Ethics Committee (INP protocol 039/2018) in accordance with the Declaration of Helsinki. All the glioma tumors had novo origin and did not receive chemotherapy before surgery. The tumor sample was resected, a portion was preserved for this study, and the other portion was submitted to the pathology service to identify the tumor grade. The characteristics of the pediatric glioma samples are given in Table 3. The classification was performed according to the World Health Organization (CNS WHO grade 1, 2, 3 and 4).

### 2.5. Extraction of Total RNA and cDNA Synthesis

The total RNA of HMC3 cell line was purified using the TRIzol method (Invitrogen, Carlsbad, CA, USA), following the manufacturer’s instructions. The quality of RNA was assessed at 260/280 nm and the integrity was evaluated on 2.0% (*w*/*v*) agarose gel electrophoresis. The samples were treated with 1 U of DNase I enzyme (Thermo Fisher Scientific, Waltham, MA, USA). The cDNA was synthesized using oligo (dT) 18 primers (Thermo Fisher Scientific), and RevertAid reverse transcriptase (Thermo Fisher Scientific). The brain glioma biopsies was disrupted using the TissueLyser system (Qiagen, Valencia, CA, USA) for 60 s at 25 Hz. Total RNA extraction and cDNA synthesis was performed as mentioned above. All the synthesized cDNAs were quantified and stored at −70 °C until use.

### 2.6. Quantitative RT-qPCR Analysis

The amplification of the specific PCR products of the sixteen genes proposed in this study (Table 1) was determined using the Fast SYBR^®^ Green Master Mix kit (Applied Biosystems, Foster City, CA, USA) in the StepOnePlusTM Real-Time PCR Systems platform (Life Technologies, Foster City, CA, USA), which was carried out according to the MIQE guidelines [14]. The RT-qPCR was performed in triplicate with the following conditions: 95 °C for 30 s, followed by 40 cycles of 95 °C for 30 s and 60 °C for 30 s. A reaction without the template was run in parallel for all plates to verify the purity of measurement within each experiment. The amplification efficiency of each primer pair was evaluated by the standard curve method using serial dilutions of cDNA (initial concentration, 100 ng) to obtain the correlation coefficient and slope values. Each run was completed with a melting curve analysis in the range of 60–95 °C.

### 2.7. Analysis of Gene Expression Stability

To determine of expression stability of the sixteen candidate genes in this study, the cycle threshold (C_T_) values for each reference gene previously obtained from RT-qPCR were analyzed with four statistical algorithms for the evaluation and selection of reference genes: BestKeeper [23], the comparative ΔC_T_ method [24], geNorm [25], and NormFinder [26]. The stability values (M) were calculated for each candidate gene using the NormFinder and geNorm software. Besides, we used the RefFinder algorithm [27], which integrates the results from BestKeeper, the comparative ΔC_T_ method, geNorm, and NormFinder, and calculates the geometric mean (geomean) for each reference gene to give the ranking index of stability [27].

### 2.8. Analysis of Relative Gene Expression in Human Glioma Samples

We evaluated the expression levels of the selected metabolic gene profile in HMC3 cells and glioma samples using the best reference gene according to the stability value obtained previously. The relative change in the gene expression of the target genes (*HK1, PFKM*, *TPI1*, *GAPDH*, *LDHAL6A*, *G6PD*, *PGD1*, *TKT1*, *SDHB*, *IDH1*, *FASN*, *ACACA*, and *ELOVL2*) was analyzed using the 2^−ΔΔCt^ method, employing the *PKM* reference gene for normalization [24]. The relative change in expression upon normalization was evaluated using ANOVA and the Tukey–Kramer test. The curve was fitted with GraphPad Prism 9 software (GraphPad Software Inc., La Jolla, CA, USA) and a statistical analysis was performed. Five replicates were included for each human glioma sample, and all reactions were run by triplicate.

## 3. Results

### 3.1. Determination of the Specificity and Efficiency of Primer Pairs

The specificity analysis of the primers for all the proposed genes was evaluated by endpoint PCR. The obtained PCR products are shown in Figure S2, for the *HK1*, *PFKM*, *TPI1*, *GAPDH*, *GAPDH^a^*, *GAPDH^b^*, *PKM*, *G6PD*, *TKT1*, *SDHB*, *FASN*, *ACACA*, and *TBP* genes, a single band of the expected size was obtained, indicating that these primers did not form primer-dimers and nonspecific amplification products. The expected amplification products were not obtained regarding the genes *LDHAL6A*, *PGD1*, and *EL0VL2* (Figure S2, lines 7, 9, and 14, respectively).

**Figure 1:**
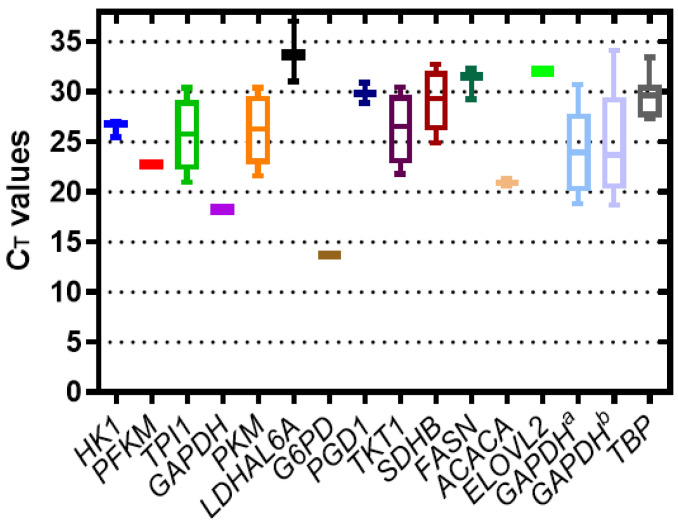
Expression levels of the sixteen candidate reference genes in the HMC3 microglial cell line (ATCC^®^ CRL-3304™). The variation is shown as median values and 25th to 75th percentiles (boxes), with whisker plots indicating the lower and upper values of C_T_ of the candidate reference genes. The values of three biological replicates are shown. The graph was plotted with the GraphPad Prism 9 software.

The transcriptional levels of the sixteen candidate reference genes were determined using RT-qPCR to compare the levels of mRNA of each gene. As seen in Figure 1, the C_T_ values for the *TPI1*, *PKM*, *TKT1*, *SDHB*, *FASN*, *GAPDH^a^*, *GAPDH^b^*, and *TBP* genes have a normal distribution according to the method of Kolmogorov and Smirnov. The *G6PD* gene showed the lowest C_T_ value (14 cycles), followed by the *PFKM*, *GAPDH*, and *ACACA* genes, with values between 17 and 23 cycles. Finally, the least-expressed genes were *HK1*, *TPI1*, *PKM*, *LDHAL6A*, *PGD1*, *TKT1*, *SDHB*, *FASN*, *ELOV2*, *GAPDH^a^*, *GAPDH^b^*, and *TBP* with C_T_ values between 26 and 34 cycles.

To confirm the specificity of all the primers, melt curves analyses were performed for the sixteen genes. In Figure S3, the melting curves obtained for the analyzed genes are shown, and a single specific peak was obtained for the genes *TPI1*, *GAPDH*, *PKM*, *G6PD*, *TKT1*, *SDHB*, *ACACA*, *GAPDH^a^*, *GAPDH^b^*, and *TBP*. The presence of a single peak in the curves indicates the alignment specificity and thereby confirms that the primers are suitable to be used in amplification reactions to obtain the desired products under the predicted alignment temperature. However, for the genes *HK1*, *PFKM*, *LDHAL6A*, *PGD1*, *FASN*, and *ELOVL2*, we observed more than one peak, apparently due to the dimer primers, so these last genes were not suitable for the analysis of gene expression. The temperature of dissociation (Tm) value for the RT-qPCR products from the genes evaluated was between 75 °C (*TBP*) and 83 °C (*TPI1*). With respect to the control without nucleic acid (NTC), no signal was detected, which indicates the absence of contamination.

Thus, we selected *TPI1*, *PKM*, *GAPDH*, *G6PD*, *SDHB*, *TKT1*, *ACACA*, *GAPDH^a^*, *GAPDH^b^*, and *TBP* as the final candidate reference genes. Next, we evaluate their expression stability to select the appropriate reference gene to measure the gene expression levels in brain glioma biopsies.

### 3.2. Stability of Candidate Reference Genes

To identify the best reference gene, we used as criteria a stable expression and minimum variability in HMC3 cells. Whereby, the expression stability of the ten final candidate reference genes was analyzed with the BestKeeper program. This algorithm calculates the standard deviation (SD) of the C_T_ values and the coefficient variance (CV) for each gene. Genes with SD values > 1 are considered unstable and therefore are not suitable to be used as reference genes [23]. The results obtained with BestKeeper are shown in Table 4 (Figure S4) and suggest that the *PKM*, *TPI1*, *GAPDH*, and *GAPDH^a^* genes are the best candidates, as they showed less variation according to the calculated deviation (lower dispersion C_T_), while the *TKT1*, *SDHB*, *G6PD*, *ACACA*, *GAPDH^b^*, and *TBP* genes had higher dispersion values.

We also used the comparative ΔC_T_ method, which compares the relative expression by pairs of genes within each sample to identify the genes suitable for expression studies. The comparation will provide information on which genes show less variability and a more stable expression among the genes analyzed [24]. The results obtained are shown in Table 4, and we observed too that the *PKM*, *TPI1*, *GAPDH*, and *GAPDH^a^* genes showed a relatively stable expression. Regarding the analysis with the geNorm program a stability value M for a particular gene; a low value of M indicates a high stability in the expression, the M values calculated for the eleven candidate reference genes are shown in Table 4. The *PKM*, *TPI1*, *GAPDH*, *GAPDH^a^*, *TKT1* and *SDHB* genes are the most stable reference genes, with M values of 0.017, 0.017, 0.035, 0.053, 0.768 and 1.446, respectively, while the *G6PD*, *ACACA*, *GAPDH^b^*, and *TBP* genes showed M values above 1.5, which is the limit suggested by the geNorm program [25]. Besides this, candidate reference genes were analyzed with the NormFinder software. This program uses a model-based algorithm to measure variation in expression between subgroups of samples [26]. The results obtained with NormFinder were similar to those of the geNorm method; both methods ranked *PKM*, *TPI1*, *GAPDH*, *GAPDH^a^*, *TKT1* and *SDHB* as among the six most stable reference genes (Table 4).

Finally, the expression stability results obtained with the four major algorithms (BestKeeper, the comparative ΔC_T_ method, geNorm, and NormFinder) (Table 5) were integrated using the RefFinder tool; this algorithm integrates the results based on the classification of each of the programs and calculates the geometric means (Geomean) for the final classification [27]. Table 5 showed the results obtained with RefFinder tool; the overall results of this analysis indicate that the *TPI1*, *PKM* and *GAPDH* genes are the most stably expressed and are thus suitable to be used as reference genes in gene expression studies.

### 3.3. Evaluation of Gene Expression Levels in Brain Glioma Biopsies

After validating and selecting the most appropriate reference genes to measure the gene expression levels, we found that the most suitable genes were *PKM*, *TPI1*, and *GAPDH*. Therefore, the *PKM* gene was used as a reference gene to measure the expression levels of genes of metabolic pathways in brain glioma biopsies of pediatric patients diagnosed with different CNS WHO grades of gliomas, because it showed the best stability ranking in the statistical analyses.

**Figure 2:**
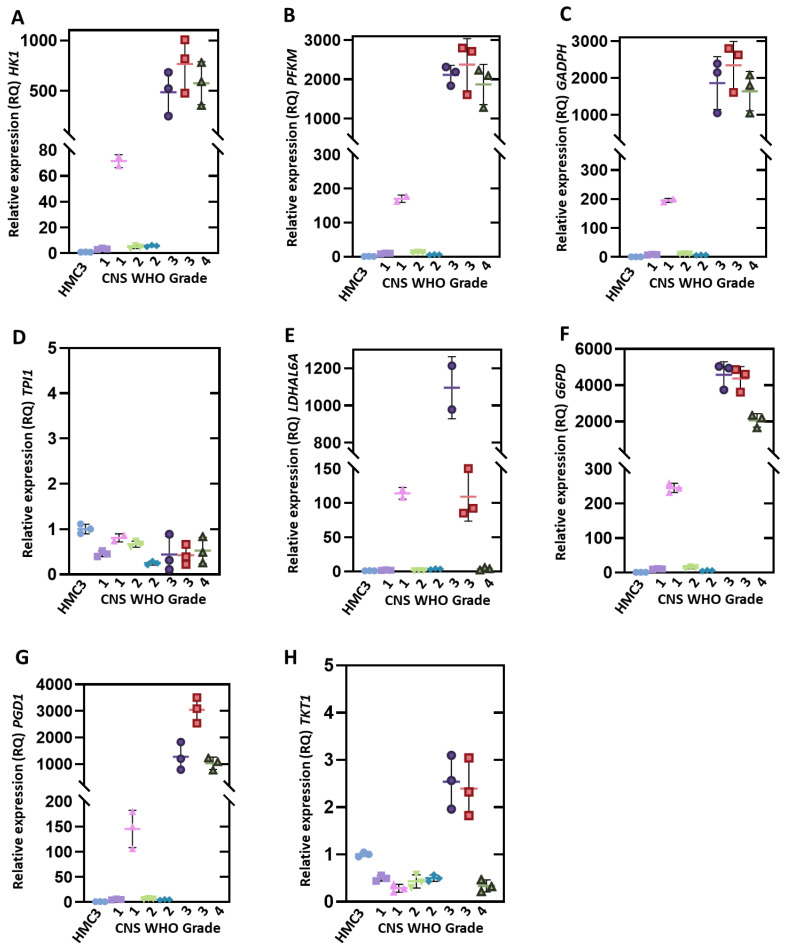
Differential expression of genes involved in essential metabolic pathways of glioma cells. (**A**–**E**) represent expression levels of the *HK1*, *PFKM*, *TPI1*, *GAPDH*, and *LDHAL6A* genes involved in glycolysis. (**F**–**H**) represent the expression levels of the *G6PD*, *PGD1*, and *TKT1* genes involved in pentose phosphate. Relative expression levels were determined using the *PKM* gene as a reference. Values are means ± SD of quadruplicate determinations. 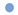 HMC3, 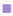 CNS WHO 1 (T1), 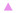 CNS WHO 1 (T12), 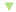 CNS WHO 2 (T4), 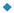 CNS WHO 2 (T18), 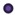 CNS WHO 3 (T7), 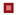 CNS WHO 3 (T10), 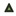 CNS WHO 4 (T9).

To investigate the first limiting step in the glucose metabolism and subsequent steps, we analyzed the expression levels of genes that encode glycolytic enzymes such as hexokinase isoform 1 (*HK1*), phosphofructokinase muscle (*PFKM*), glyceraldehyde-3-phosphate dehydrogenase (*GAPDH*), triosephosphate isomerase 1 (*TPI1*), and lactate dehydrogenase (*LDHAL6A*). We found that the *HK1*, *PFKM*, *GAPDH*, and *LDHAL6A* genes were overexpressed in all the clinical samples analyzed compared with the HMC3 microglia cell line (Figure 2). Moreover, the high expression of *HK1*, *PFKM*, and *GAPDH* genes correlated with the tumor degree of malignancy. Besides this, we observed that, in CNS WHO grade 1 and 2 samples, the expression levels of the *HK1*, *PFKM*, and *GAPDH* genes ranged from 2 to 65, 11 to 163, and 10 to 190-fold increases, respectively, vs. HMC3. Meanwhile, in CNS WHO grade 3 and 4 gliomas, the levels of expression ranged from 486 to 767, 2097 to 2713, and 1798 to 2628-fold increases, respectively (Figure 2A–C). The *LDHAL6A* gene showed overexpression in all the clinical samples analyzed (Figure 2E). Regarding the *TPI1* gene, we found that it was not overexpressed in any clinical sample of pediatric glioma (Figure 2D).

We also evaluated the expression levels of the genes involved in PPP, such as *G6PD*, *PGD1*, and *TKT1* (Figure 2). The results obtained show that the gene encoding the G6PD enzyme, which is the first enzyme in the pathway and controls the flow of the PPP, showed overexpression in all clinical samples (Figure 2F). Moreover, this overexpression is related to the grade of the tumor; the levels of expression of *G6PD* were in the range of 5 to 245-fold in CNS WHO grade 1 and 2 gliomas compared to CNS WHO grade 3 and 4 gliomas, with a range of 2197 to 4946-fold. The same pattern was observed in the *PGD1* gene (Figure 2G), which showed an expression level of 5 to 150-fold in the CNS WHO grade 1 and 2 glioma samples, compared to 4946-fold in the CNS WHO grade 3 and 4 glioma samples. Finally, when we analyzed the *TKT1* gene, we found that this gene was not overexpressed in CNS WHO grade 1 and 2 samples. However, in CNS WHO grade 3 samples, an overexpression of 2.3–2.5-fold was found, but in CNS WHO grade 4 samples, it was not overexpressed (Figure 2H). However, this last result is important for corroborating the analysis of more clinical samples of brain glioma biopsies (CNS WHO grade 4).

**Figure 3:**
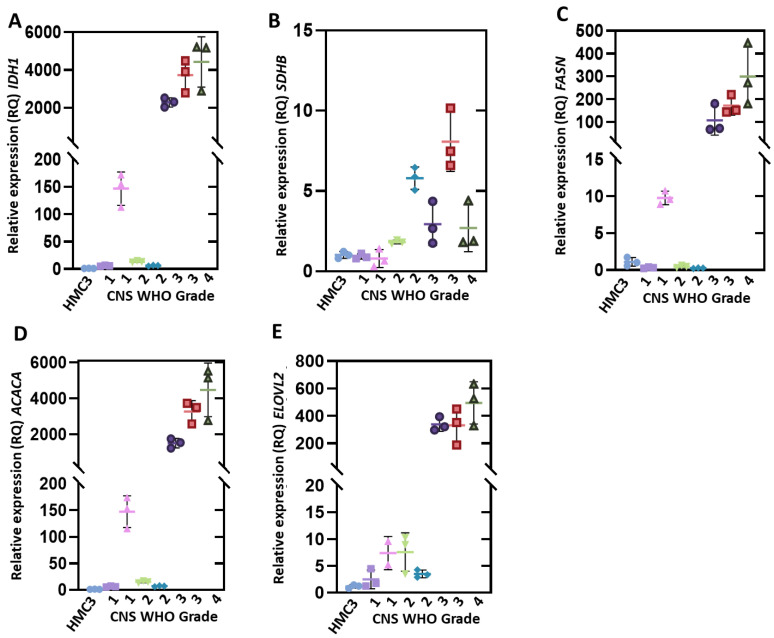
Expression levels of genes that encode enzymes that participate in the essential metabolic pathways of glioma cells. (**A**,**B**) represent the expression levels of IDH1 A and SDHB genes involved in the Krebs cycle. (**C**–**E**) represent the expression levels of the FASN, ACACA, and ELOVL2 genes involved in the fatty acid synthesis genes. Relative expression levels were determined using the PKM gene as a reference. Values are means ± SD of quadruplicate determinations. 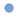 HMC3, 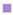 CNS WHO 1 (T1), 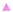 CNS WHO 1 (T12), 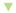 CNS WHO 2 (T4), 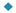 CNS WHO 2 (T18), 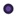 CNS WHO 3 (T7), 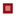 CNS WHO 3 (T10), 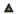 CNS WHO 4 (T9).

Then, we evaluated the genes that code for two enzymes of the Krebs cycle (*IDH1* and *SDHB*). As seen in Figure 3A, we found that the *IDH1* gene was overexpressed in all clinical samples, and the expression level correlates with the grade of the tumor. In CNS WHO grade 3 and 4 tumors, we found a greater overexpression 5 to 155-fold in CNS WHO grade 1 and 2 gliomas and 2305 to 5167-fold in CNS WHO grade 3 and 4 gliomas. With respect to the *SDHB* gene (Figure 3B), we found that in CNS WHO grade 1 glioma it was not overexpressed. In CNS WHO grade 2–4 samples an increase in the expression of this gene was observed, ranging from 1.7 to 5.8-fold for samples of grade 2, 2.6 to 7.4-fold for grade 3 samples, and 1.8-fold for grade 4 samples.

Finally, the *FASN*, *ACACA*, and *ELOVL2* genes, which participate in the fatty acid synthesis pathway, were evaluated. As shown in Figure 3C, it was observed that the *FASN* gene was not overexpressed in the CNS WHO grade 1 and 2 samples, while in the CNS WHO grade 3 and 4 samples an overexpression of the gene was observed from 72 to 180 -fold. Regarding the *ACACA* and *ELOVL2* genes, we observed that these two genes were overexpressed in all glioma samples, and there was a relationship between the degree of tumor and expression. For the *ACACA* gene, an overexpression of 6 to 153-fold was found in CNS WHO grade 1 and 2 gliomas, while the expression levels of CNS WHO grade 3 and 4 gliomas showed 1534 to 5133-fold increases with respect to HMC3 (Figure 3D). In the case of the *ELOVL2* gene, the level of overexpression was 1.4 to 9.5-fold in CNS WHO grade 1 and 2 samples and 337 to 353-fold in CNS WHO grade 3 and 4 samples (Figure 3E).

## 4. Discussion

The RT-qPCR technique has become the method of choice for gene expression due to its wide range of quantification in biological samples, and it also shows a high sensitivity and precision. However, due to the extreme sensitivity of the RT-qPCR technique, an adequate normalization of the results for the expression levels of the genes is required. For this reason, the use of an internal control called a reference gene is required, which ideally must show stable expression levels, and it must not vary significantly between samples and experimental conditions [28,29]. Although there are reports of reference genes being validated for expression studies in human gliomas, it is not possible to establish a consensus on this issue. For example, Valente et al. [17] reported that the *TBP* and *HPRT1* genes are suitable as references for gene expression studies in clinical samples of glioblastoma. Another study found that the *YWHAZ* gene [18] is a suitable reference gene; later, the *RPL13A* and *TBP* genes were also proposed by Aithal et al. [19] as reference genes. In the present study, we proposed to evaluate a profile of metabolic genes as reference genes, including *HK1*, *PFKM*, *TPI1*, *GAPDH*, *PKM*, *LDHAL6A*, *G6PD*, *PGD1*, *TKT1*, *SDHB*, *FASN*, *ACACA*, and *ELOVL2*, because these genes are required for the maintenance of basic cellular functions that are important for the existence of any cell type, as they express proteins that are essential for the viability of the cells. In this context, we considered it important to perform an analysis to validate and select reference genes for studies of expression levels in brain glioma biopsies.

Surprisingly, our results showed that *GAPDH*, which is commonly used as a reference gene in gene expression studies, is not the most stable housekeeping gene in HMC3 cells. It is interesting to note that our results agreed with other reports, where it was also found that the *GAPDH* gene is not the most suitable for use as an internal control gene in cancer studies [30]. Moreover, the analysis that we realized using Bestkeeper, the comparative ΔC_T_ method, geNorm, NormFinder and RefFinder to calculate a final classification of candidate genes for validation as reference genes allowed us to propose the following genes (from most to least stable): *PKM* > *TPI1* > *GAPDH* > *GAPDH^a^* > *TKT1* > *SDHB* > *G6PD* > *ACACA* > *PGD1* > *GAPDH^b^ > TBP* (Table 4 and Table 5). Thus, we propose *PKM* and *TPI1* as suitable reference genes in gene expression studies in brain glioma biopsies.

The results show that it is not easy to identify a universal reference gene that possesses all the ideal characteristics. In the initial endpoint PCR analysis of the sixteen genes, three genes were not amplified (*LDHAL6A*, *PGD1*, and *ACACA2*). Later, in the validation of the RT-qPCR assays, some of them were found to be unspecific and/or inefficient (*HK1*, *PFKM*, *PGD1*, *FASN*, *ACACA*, and *ELOVL2*). This highlights the importance of selecting appropriate reference genes for mRNA quantification by RT-qPCR, because the use of non-validated reference genes can lead to questionable results and errors in data interpretation [31].

**Figure 4:**
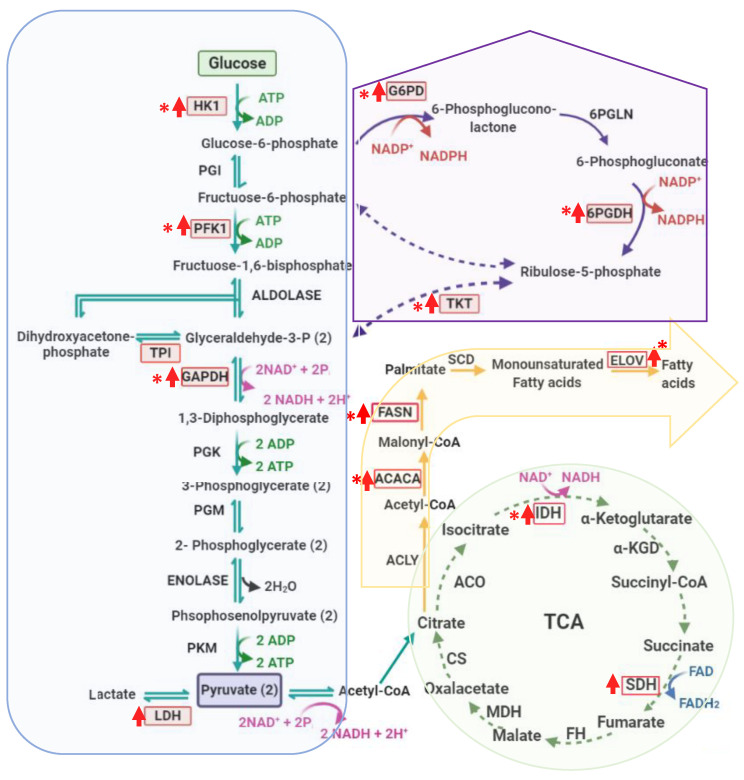
Metabolic routes relevant to this study. The genes analyzed in this study are shown in a red box, red arrows indicate genes that showed overexpression, and an asterisk represents those genes that showed an increase in the level of expression regarding the CNS WHO grade of the brain glioma biopsies. The gliomas undergo metabolic remodeling to meet the needs of the highly proliferative cells, which can be summarized as an increase in the expression of some genes involved in glycolysis, such as *HK1*, *PFKM*, *GAPDH*, and *LDHAL6A*. Besides this, the overexpression of some genes involved in PPP was also demonstrated (*G6PD*, *PGD1*, and *TKT1*), which will probably allow the glioma cells to have a more significant enzymatic activity in this pathway, and thus the cells obtain precursors for nucleotide synthesis and reducing power. Finally, the Krebs cycle and fatty acid synthesis pathways play central roles in cellular metabolism; we also found that the *IDH1*, *SDHB*, *FASN*, *ACACA*, and *ELOVL2* genes were overexpressed, resulting in significant disturbances in the cellular metabolism of gliomas.

Metabolic reprogramming is a hallmark of cancer [32], and the Warburg effect is a characteristic phenotype of this reprogramming, allowing a rapid generation of useful intermediates in the biosynthesis of macromolecules. It has been reported that glioma cells overexpress glucose transporters such as *GLUT1* and *GLUT3*, allowing them to increase the flux of glucose towards glycolysis [33]. Due to this, we decided to analyze the expression levels of some glycolytic genes in pediatric glioma samples of different grades, and found that the *HK1*, *PFKM*, *GAPDH*, and *LDHAL6A* genes are overexpressed in human brain glioma biopsies. Furthermore, our study also demonstrated that an increase in *HK1*, *PFKM*, and *GAPDH* expression could be associated with the CNS WHO grading of gliomas. For this reason, the *HK1*, *PFKM*, and *GAPDH* genes could be essential for the progression of glioma to malignant grades, and in this way, they could be useful biomarkers and even targets for the therapy of gliomas. These results also suggest that gliomas have a high glycolytic flux (Figure 4). One of the enzymes that determines the pyruvate’s final fate is LDH, an enzyme that catalyzes the conversion of pyruvate to lactate. In the analyzed samples, we found an overexpression of the *LDHAL6A* gene in all samples, regardless of the CNS WHO grading, which is an agreement with the Warburg effect in glioma cells. Besides this, our results agree with other studies where glioma cells have shown high glycolytic levels compared to healthy brain tissue [34,35]. These results imply that glioma cells catabolize glucose in the glycolysis pathway, generating lactate as the final product (Figure 4). Regarding the *TPI1* gene, we found that it was not overexpressed in any clinical sample of pediatric glioma. The TPI enzyme catalyzes the isomerization of dihydroxyacetone phosphate to glyceraldehyde-3-phosphate (G3P) to continue the glycolytic pathway. However, the glioma cells could obtain G3P from the PPP, and thus, the TPI enzyme expression did not increase (Figure 4). Furthermore, TPI is a highly efficient enzyme, thus the rate with which it catalyzes the isomerization of G3P is only limited by the diffusion of the substrate [36].

The expression level of the *G6PD* gene, which encodes the enzyme that controls the pentose phosphate pathway´s flow, was also analyzed. A higher level of overexpression was found in CNS WHO grade 3 and 4 gliomas compared to CNS WHO grade 1 and 2 gliomas. We found the same pattern in the expression level of the *PGD1* gene, which suggests that PPP is also an important pathway for pediatric glioma cells, since PPP provides the pentoses phosphate necessary for nucleic acid synthesis in the cancer cells (Figure 4), allowing rapid growth and proliferation, characteristics of CNS WHO grade 3 and 4 gliomas [37]. Furthermore, PPP supplies NADPH to the cell, which is an important molecule in the synthesis of lipids and the protection of cells under conditions of oxidative stress [38]. These results suggest the importance of PPP in the brain glioma biopsies analyzed.

Another important finding in the present work is that the *TKT1* gene is not overexpressed in CNS WHO grade 1 and 2 gliomas, while in CNS WHO grade 3 and 4 gliomas, it shows an increase in expression, which correlates well with the CNS WHO disease grade (Figure 3). It is important to mention that these results agree with previous studies that have reported the overexpression of enzymes of the PPP in human cancer tissues and could be useful as a marker of malignancy in gliomas [39,40,41].

Besides this, genes that code for Krebs cycle proteins such as *IDH1* and *SDHB* were also analyzed. The *IDH1* and *SDHB* genes are both involved in the fundamental processes of the production of reducing equivalents in the form of reduced adenine dinucleotide phosphate (NADPH) and reduced flavin adenine dinucleotide (FADH_2_), which are oxidized in the electron transport chain (ETC) to produce adenosine triphosphate (ATP) [42]. Regarding the *IDH1* gene, we found that it is overexpressed in all clinical samples and correlates with the grade of the tumor. In the process of cancer development, the metabolic activity of cancer cells is increased; consequently, an increase in intracellular ROS is observed, related to a wide spectrum of activities [43]. To prevent toxic levels of ROS occurring, cancer cells increase flux through the metabolic pathways that produce NADPH to meet the demands of this molecule; therefore, glioma cells probably show a greater overexpression of the *IDH1* gene in CNS WHO grade 3 and 4 gliomas.

The SDH enzyme has been classically considered to be a mitochondrial enzyme with the unique characteristic of participating in both the Krebs cycle and ECT. Furthermore, several studies have highlighted the role of succinate in biological processes other than metabolism, with tumorigenesis being the most prominent [44]. For this reason, succinate has been defined as an oncometabolite, as well as fumarate, and the *SDHB* gene has been identified as a tumor suppressor, with even alterations in SDH activity leading to succinate accumulation. Various reports have shown that the expression levels of *SDH* and succinate and fumarate production can be altered in cancer cells [45,46]. Our results show that in CNS WHO grade 1 glioma samples there is no overexpression of *SDHB*, meanwhile the samples of CNS WHO grade 2 showed an expression level of 1.7 and 5.7-fold, and CNS WHO grade 3 and 4 samples showed overexpression of 2.6 to 7.47-fold. Besides this, the level of overexpression of the *SDHB* gene is lower compared with that of the *IDH1* gene. The lowest expression of *SDHB* probably causes a decrease in the amount of SDHB enzyme translated, inducing an altered metabolic phenotype by accumulating succinate and leading to a bioenergetic shift from mitochondrial respiration to cytosolic glycolysis [47,48]. It has been reported that the silencing of *SDHB* in hepatocellular carcinoma cell lines leads to an alteration in energy metabolism and an almost complete loss of mitochondrial membrane potential, as well as a decrease in the expression of Complex III and IV of oxidative phosphorylation, causing an increase in the acidity of the medium, an increase in glucose uptake, and an overexpression of hexokinase 1 (*HK1*) [48], which is in agreement with the *HK1* overexpression found in the present work.

Besides, when we analyzed relevant genes in the fatty acid synthesis pathway, we found that the *FASN* gene was overexpressed in CNS WHO grade 3 and 4 glioma samples in the range of 72 to 180-fold. These results agree with a study performed by Tao et al. [49], where they found that the expression levels of *FASN* were higher in CNS WHO grade 3 and 4 gliomas (62-fold) than in CNS WHO grade 1 and 2 gliomas. The overexpression of *FASN* has also been reported in different human tumors, such as adenocarcinoma of the prostate, ovarian neoplasm, and thyroid [50,51,52,53]. It is important to mention that the present study is the first time that the levels of expression of the *ACACA* and *ELOVL2* genes have been analyzed in clinical samples of pediatric gliomas, and a relationship was also found between the level of overexpression and the CNS WHO grade of the glioma, indicating that these genes could also be useful biomarkers in the diagnosis and progression of gliomas.

Finally, although the research objective was to determine the differential expression levels of genes of metabolic pathways, our results should be interpreted with care. They cannot be entirely representative of the different grades of gliomas. Due to the scope of this initial research project, the variation represented in the results is limited according to the number of samples analyzed. This preliminary study involved only seven glioma tumor biopsies, and an increase in sample size being necessary to confirm the relationship between the expression levels of the metabolic genes analyzed and the CNS WHO grade glioma. Furthermore, another limitation of the study is that despite having managed to find and propose two new reference genes for expression studies in Gliomas, we performed the analysis variation of the candidate reference genes studied only in a single cell line.

## 5. Conclusions

The results obtained in this study suggest that the *PKM* and *TPI1* genes could be used as reference genes to normalize and quantify the expression of target genes in pediatric gliomas samples by RT-qPCR. This is the first report on the stability of the expression of the different candidate reference genes that participate in metabolic pathways in gliomas. The analysis of the expression levels of the genes involved in important metabolic pathways for glioma cells revealed that, in order to meet the needs of the highly proliferative cells of gliomas, they undergo metabolic remodeling, which can be summarized as an increase in glucose consumption for a higher production of glycolytic ATP and lactate as the final product, where the overexpression of genes involved in glycolysis such as *HK1*, *PFKM*, *GAPDH*, and *LDHAL6A* was observed. Besides this, the overexpression of some genes involved in the PPP was also demonstrated (*G6PD*, *PGD1*, and *TKT1*), which will probably allow the glioma cells a more significant enzymatic activity in this pathway, thus the cells obtain precursors for nucleotide synthesis and maintaining redox homeostasis. Finally, the Krebs cycle and fatty acid synthesis pathways play central roles in cellular metabolism; we also found that the *IDH1*, *SDHB*, *FASN*, *ACACA*, and *ELOV2* genes were overexpressed, resulting in significant disturbances in the cellular metabolism of gliomas.

## Figures and Tables

**Table 1:** Genes analyzed in this study.

Gen ID	GenBank	Gene Symbol	Gene Full Name	Function
3098	NM_000188.2	*HK1*	Hexokinase 1, variant 1	Transferase in glycolysis
5213	NM_000289.6	*PFKM*	Phosphofructokinase, muscle, variant 4	Transferase in glycolysis
7167	NM_000365.5	*TPI1*	Triose phosphate isomerase 1, variant 1	Isomerase in glycolysis
2597	NM_001256799.2	*GAPDH*	Glyceraldehyde-3-phosphate dehydrogenase, variant 2	Oxidoreductase in glycolysis
5315	NM_002654.6	*PKM*	Pyruvate kinase M1/2, variant 1	Transferase in glycolysis
160287	NM_001144071.1	*LDHAL6A*	Lactate dehydrogenase A like 6A, transcript variant 2	Oxidoreductase in glycolysis
2539	NM_001360016.2	*G6PD*	Glucose-6-phosphate dehydrogenase, variant 1	Oxidoreductase in pentose phosphate
5226	NM_002631.4	*PGD1*	phosphogluconate dehydrogenase, variant 1	Oxidoreductase in pentose phosphate
7086	NM_001064.4	*TKT1*	Transketolase, variant 1	Transferase in pentose phosphate
6390	NM_003000.2	*SDHB*	Succinate dehydrogenase complex iron sulfur subunit B	Oxidoreductase in Krebs’s cycle
2194	NM_004104.5	*FASN*	Fatty acid synthase	Transferase in fatty acid synthesis
31	NM_198834.3	*ACACA*	Acetyl-CoA carboxylase α, variant 1	Ligase in fatty acid synthesis
54898	NM_017770.4	*ELOVL2*	Fatty acid elongase 2	Transferase in fatty acid synthesis
6908	NM_003194	*TBP*	TATA-binding protein	General transcription factor

**Table 2:** Sequence of primers used in this study.

Gene	Sequence 5′ → 3′	Amplicon Size (pb)	Tm (°C)
*HK1*	Fw 5′-AAAGCGAGGGGACTATGA-3′ Rv 5′-ATCAATGTGCCTCAGTTCC-3′	157	60
*PFKM*	Fw 5′-AGAATCTGGTGGTTAAGCG-3′ Rv 5′-GAGGCTCACTACACAGGCT-3′	176	60
*TPI1*	Fw 5′-CGCAGATAACGTGAAGGAC-3′ Rv 5′-CAGTCACAGAGCCTCCATAA-3′	190	60
*GAPDH*	Fw 5′-CTCTGATTTGGTCGTATTGG-3′ Rv 5′-GATGACAAGCTTCCCGTT-3′	184	60
*PKM*	Fw 5′-GGTTCGGAGGTTTGATGA-3′ Rv 5′-GGCTTCTTGATCATGCTCT-3′	186	60
*LDHAL6A*	Fw 5′-GCGACTCAAGTGTTCCTGT-3′ Rv 5′-GCTAATGCCCCAAGAAGTAT-3′	179	60
*G6PD*	Fw 5′-ATATTTATGGCAGCCGAGG-3′ Rv 5′-GTCAATGGTCCCGGTGT-3′	190	60
*PGD1*	Fw 5′-CATTCGGAAGGCACTCTAC-3′ Rv 5′-CTTGTCTTCAAGGCCCAA-3′	199	60
*TKT1*	Fw 5′-GATCACGGGGGTAGAAGA-3′ Rv 5′-TGTCCCCAACTTTGTAGCT-3′	200	60
*SDHB*	Fw 5′-TTCTTATGCAGGCCTATCG-3′ Rv 5′-GGTTGCCATCATTTTCTTG-3′	185	60
*FASN*	Fw 5′-CCGCTCTGGTTCATCTG-3′ Rv 5′-GGTCTATGAGGCCTATCTGG-3′	226	60
*ACACA*	Fw 5′-AAGAGGCAATTTCAAACATG-3′ Rv 5′-ATGGTGTCAGGTCGCTC-3′	182	60
*ELOVL2*	Fw 5′-AAGCTGACATCCGGGTAG-3′ Rv 5′-TGTCCACAAGGTATCCAGTT-3′	186	60
*GAPDH^a^*	Fw 5′-AGATCCCTCCAAAATCAAGTGG-3′ Rv 5′-GGCAGAGATGATGACCCTTTT-3′	129	60
*GAPDH^b^*	Fw 5′-TGCACCACCAACTGCTTAGC-3′ Rv 5′-GGCATGGACTGTGGTCATGAG-3′	87	60
*TBP*	Fw 5′-GAGCTGTGATGTGAAGTTTCC-3′ Rv 5′-TCTGGGTTTGATCATTCTGTAG-3′	117	60

*GAPDH^a^* [17], *GAPDH^b^* [19], *TBP* [17,19].

**Table 3:** Diagnostic characteristics of the brain glioma biopsies of pediatric patients.

Code	Gender	Age	Pathology Diagnoses	CNS WHO Grade
T1	Male	10	Pylocitic astrocytoma	1
T12	Male	3	Pylocitic astrocytoma	1
T4	Female	3	Gemystocitic Astrocytoma	2
T18	Female	13	Diffuse astrocytoma	2
T7	Male	6	Stem Glioma	3
T10	Male	14	Anaplasic astrocytoma	3
T9	Female	9	Glioblastoma	4

**Table 4:** Expression stability of the ten candidate reference genes analyzed in the HMC3 microglial cell line.

Gene	BestKeeeper	Comparative ΔC_T_	geNorm	NormFinder	RefFinder (Geomean)
*TPI1*	0.024	4.29	0.017	0.009	1.41
*PKM*	0.016	4.29	0.017	0.009	1.73
*GAPDH*	0.029	4.29	0.035	0.020	2.06
*GAPDH^a^*	0.044	4.30	0.053	0.030	4.00
*TKT1*	1.40	4.83	0.768	1.689	5.23
*SDHB*	1.88	5.49	1.445	1.207	5.73
*G6PD*	3.52	6.21	2.373	5.329	7.00
*ACACA*	4.14	6.77	2.986	6.275	8.24
*GAPDH^b^*	4.88	7.84	3.477	7.396	9.49
*TBP*	12.18	16.76	6.646	16.71	11.00

**Table 5:** Expression stability ranking of the selected candidate reference genes using RefFinder.

Rank	BestKeeper	Comparative ΔC_T_	geNorm	NormFinder	RefFinder
1	*PKM*	*PKM*	*PKM*	*PKM*	*TPI1*
2	*TPI1*	*TPI1*	*TPI1*	*TPI1*	*PKM*
3	*GAPDH*	*GAPDH*	*GAPDH*	*GAPDH*	*GAPDH*
4	*GAPDH^a^*	*GAPDH^a^*	*GAPDH^a^*	*GAPDH^a^*	*GAPDH^a^*
5	*TKT1*	*TKT1*	*TKT1*	*SDHB*	*TKT1*
6	*SDHB*	*SDHB*	*SDHB*	*TKT1*	*SDHB*
7	*G6PD*	*G6PD*	*G6PD*	*G6PD*	*G6PD*
8	*ACACA*	*ACACA*	*ACACA*	*ACACA*	*ACACA*
9	*GAPDH^b^*	*GAPDH^b^*	*GAPDH^b^*	*GAPDH^b^*	*GAPDH^b^*
10	*TBP*	*TBP*	*TBP*	*TBP*	*TBP*

## Data Availability

Not applicable.

## Supplementary Materials

The following are available online at https://www.mdpi.com/article/10.3390/genes12091335/s1, Figure S1. Heat map and boxplot of the expression of the candidate reference genes based on GSE16011 database. Figure S2. Analysis of the specificity of the amplification of the candidate reference genes by endpoint PCR. Figure S3. The specificity of RT-qPCR amplification for sixteen candidate reference genes. Figure S4. Expression stability of the eleven candidate reference genes analyzed in the HMC3 microglial cell line. Table S1. Expression values of candidate reference genes.
