# Supplementary material for: Validation and Selection of New Reference Genes for RT-qPCR Analysis in Pediatric Glioma of Different Grades
Source: Genes (Basel). 2021 Aug 27;12(9):1335. doi: 10.3390/genes12091335 (PMC8468898; doi:10.3390/genes12091335)
Supplement: Supplementary file 1 [file genes-12-01335-s001.zip › genes-1319305-supplementary.pdf]

Supplementary Materials

Table S1. Expression values of candidate reference genes. Based on GSE16011 database, we showed the log2-foldchange value of the selected reference genes. Hexokinase (*HK1*), Phosphofructokinase (*PFKM*), Table 1. Glyceraldehyde 3 phosphate dehydrogenase (*GAPDH*), Pyruvate kinase (*PKM*), Lactate dehydrogenase (*LDHAL6A*), Glucose 6 phosphate dehydrogenase (*G6PD*), Phosphogluconate dehydrogenase (*PGD1*), Transketolase (*TKT1*), Succinate dehydrogenase (*SDHB*), Fatty acid synthase (*FASN*), Acetyl Co-A carboxylase (*ACACA*), Elongase fatty acid (*ELOVL2*), Glyceraldehyde 3 phosphate dehydrogenase (*GAPDH*[a]), TATA-binding protein (*TBP*).

Gene	**ID**	**adj.P.Val**	**P.Value**	**logFC**
HK1	3098	4.82E-08	4.12E-09	-0.9937336
PFKM	5213	4.37E-02	1.93E-02	-0.4858195
TPI1	7167	9.55E-01	9.33E-01	-0.0176529
GAPDH	2597	9.15E-01	8.78E-01	0.013981
PKM	5315	7.27E-01	6.40E-01	-0.1173536
LDHAL6A	160287	5.23E-01	4.12E-01	-0.050123
G6PD	2539	2.82E-01	1.87E-01	-0.2256547
PGD1	5226	4.18E-03	1.23E-03	0.6367549
TKT1	7086	6.29E-01	5.25E-01	0.1378224
SDHB	6390	3.45E-01	2.41E-01	-0.1845842
FASN	2194	1.59E-02	5.80E-03	-0.3050502
ACACA	31	3.36E-02	1.41E-02	-0.5131175
ELOVL2	54898	5.31E-01	4.20E-01	0.4873548
TBP	6908	8.85E-03	2.95E-03	0.4248779

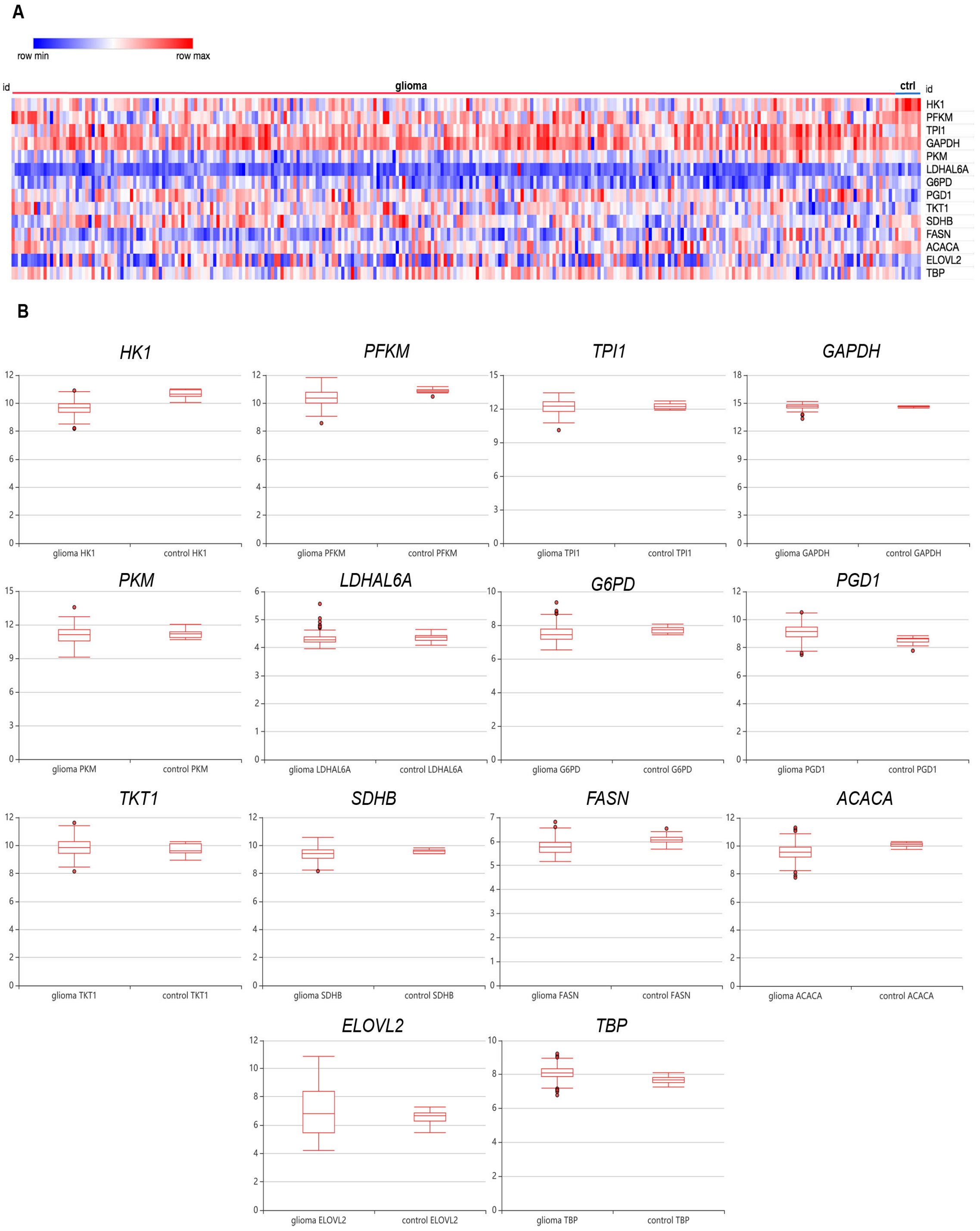

Figure S1. Heat map (A) and boxplot (B) of the expression of the candidate reference genes based on GSE16011 database. This database includes 276 glioma samples and 8 controls.

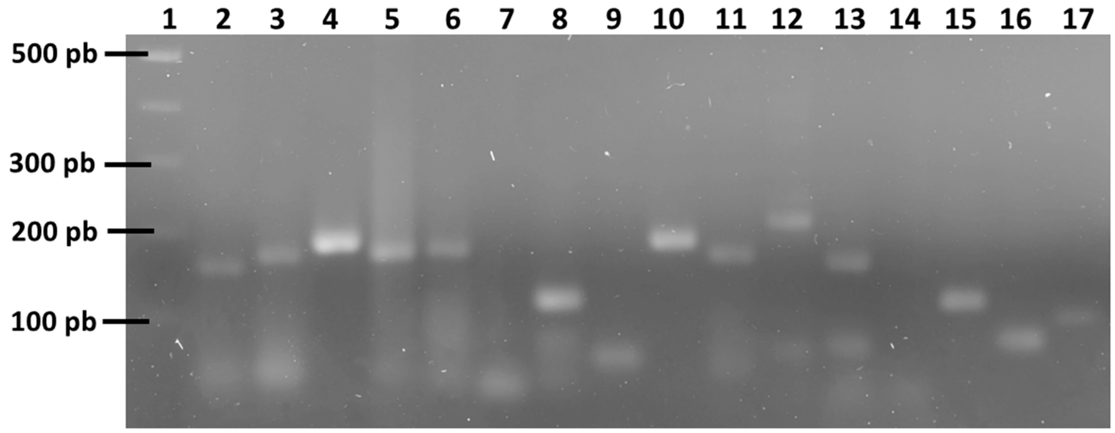

Figure S2. Analysis of the specificity of the amplification of the candidate reference genes by endpoint PCR**.** Amplified fragments obtained were visualized on 2% agarose gels. Line 1: GeneRuler 1Kb Plus DNA molecular weight marker (Termo Scientifc®). The gel was stained with Midori Green Advance. 2: Hexokinase (*HK1*). 3: Phosphofructokinase (*PFKM*). 4: Triosephosphate isomerase (*TPI1*). 5: Glyceraldehyde 3 phosphate dehydrogenase (*GAPDH*). 6: Pyruvate kinase (*PKM*). 7: Lactate dehydrogenase (*LDHAL6A*). 8: Glucose 6 phosphate dehydrogenase (*G6PD*). 9: Phosphogluconate dehydrogenase (*PGD1*). 10: Transketolase (*TKT1*). 11: Succinate dehydrogenase (*SDHB*). 12: Fatty acid synthase (*FASN*). 13: Acetyl Co-A carboxylase (*ACACA*). 14: Elongase fatty acid (*ELOVL2*). 15: Glyceraldehyde 3 phosphate dehydrogenase (*GAPDH*[a]). 16: Glyceraldehyde 3 phosphate dehydrogenase (*GAPDH*[b]). 17: TATA-binding protein (*TBP*).

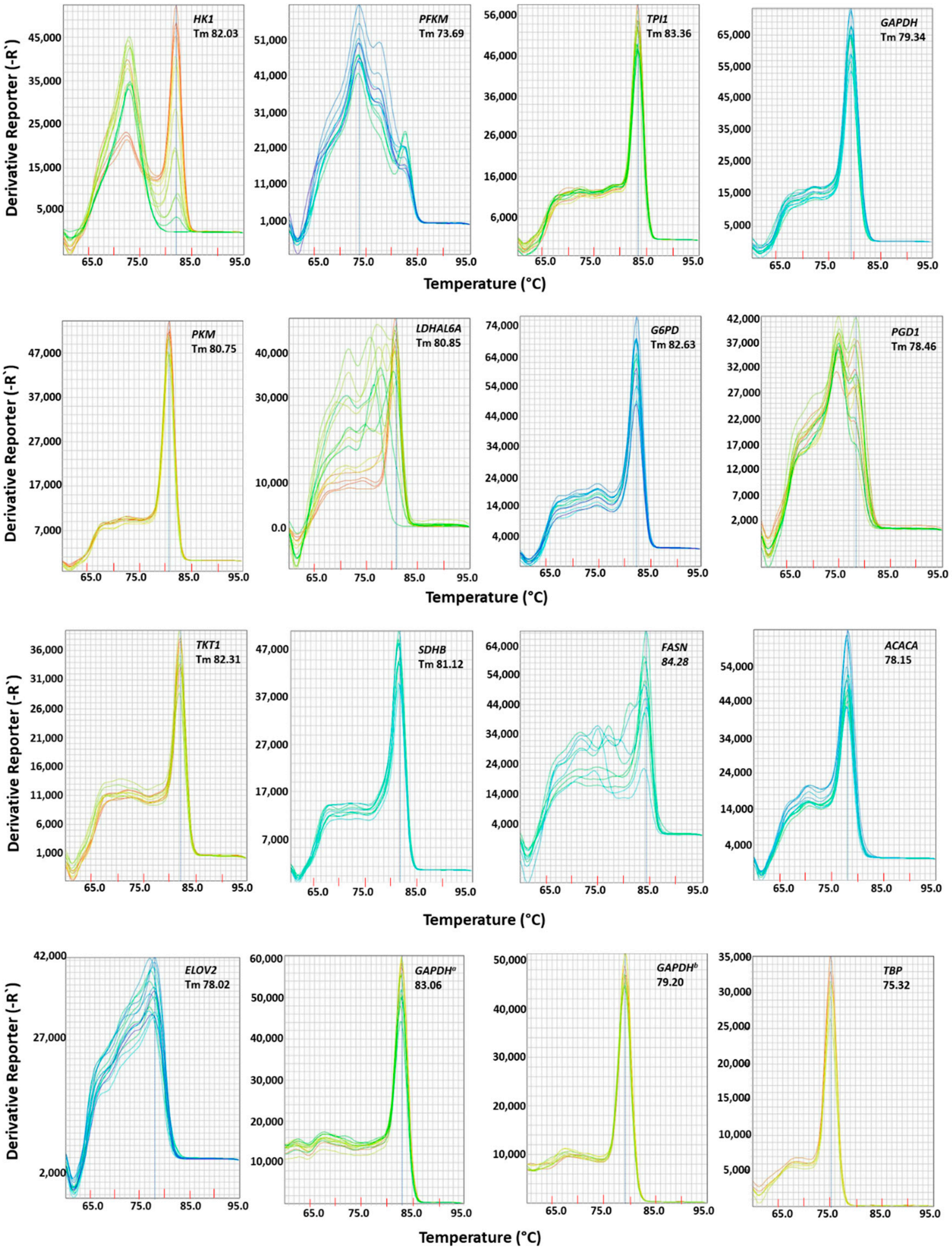

Figure S3. The specificity of RT-qPCR amplification for sixteen candidate reference genes. Melting curves were determined after completing the amplification cycles, and the reactions were subjected to a temperature gradient from 60 to 95 °C. The detection was performed using SYBR green as the intercalating dye, and each of the experiments was performed with 4 independent replicates. The Tm values of the RT-qPCR products are shown in the graphs of the corresponding genes.

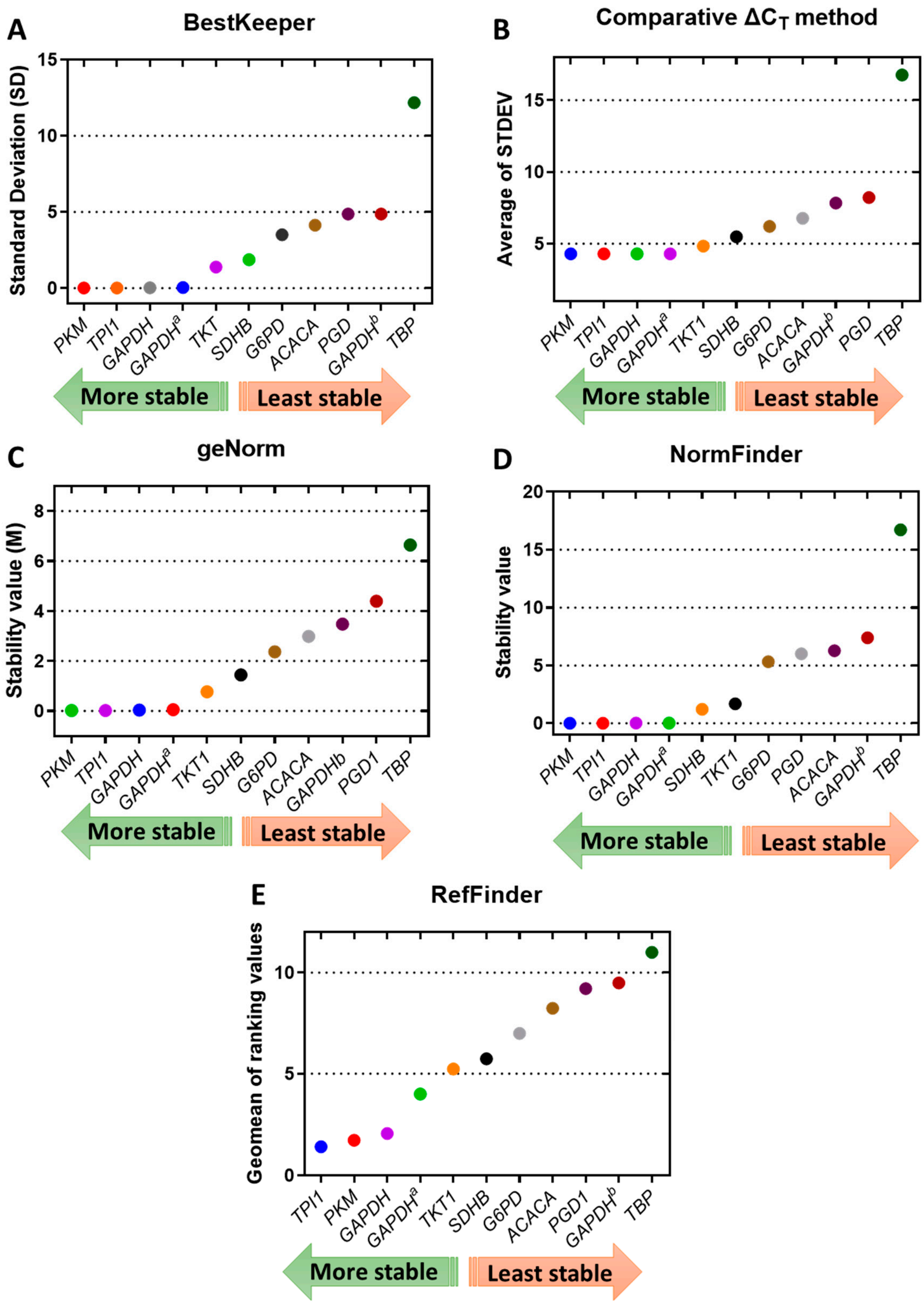

Figure S4. Expression stability of the eleven candidate reference genes analyzed in the HMC3 microglial cell line. The expression levels were calculated by the BestKeeper (A), comparative ΔC_T method (B), geNorm (C), NormFinder (D), and RefFinder (E) software.
